# Control of fluid intake by estrogens in the female rat: role of the hypothalamus

**DOI:** 10.3389/fnsys.2015.00025

**Published:** 2015-03-04

**Authors:** Jessica Santollo, Derek Daniels

**Affiliations:** Department of Psychology, University at Buffalo SUNYBuffalo, NY, USA

**Keywords:** thirst, salt appetite, hypothalamus, estrogen receptor, estrogens, drinking behavior, body fluid regulation

## Abstract

Body fluid homeostasis is maintained by a complex network of central and peripheral systems that regulate blood pressure, fluid and electrolyte excretion, and fluid intake. The behavioral components, which include well regulated water and saline intake, are influenced by a number of hormones and neuropeptides. Since the early 1970s, it has been known that the ovarian estrogens play an important role in regulating fluid intake in females by decreasing water and saline intake under a variety of hypovolemic conditions. Behavioral, electrophysiological, gene and protein expression studies have identified nuclei in the hypothalamus, along with nearby forebrain structures such as the subfornical organ (SFO), as sites of action involved in mediating these effects of estrogens and, importantly, all of these brain areas are rich with estrogen receptors (ERs). This review will discuss the multiple ER subtypes, found both in the cell nucleus and associated with the plasma membrane, that provide diversity in the mechanism through which estrogens can induce behavioral changes in fluid intake. We then focus on the relevant brain structures, hypothesized circuits, and various peptides, such as angiotensin, oxytocin, and vasopressin, implicated in the anti-dipsogenic and anti-natriorexigenic actions of the estrogens.

## Introduction

**Figure 1:**
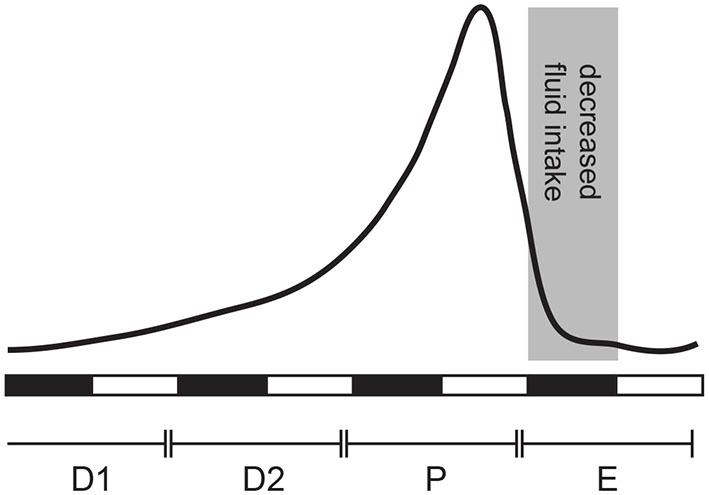
**Estradiol (E2) secretion across the estrous cycle in the female rat**. E2 levels are low during diestrus 1 (D1), begin to rise at the end of diestrus 2 (D2), peak in late proestrus (P), and drop to baseline levels during estrus (E). Behavioral changes associated with estrus occur during the dark phase, referred to here as “behavioral estrus” (shaded area).

Cardiovascular disease is the leading cause of death in women over the age of 65 (Go et al., 2013). Because a woman’s risk of cardiovascular disease increases around the age of menopause, it suggests that ovarian hormones provide a protective role (Reckelhoff, 2004; Rosano et al., 2007; Yang and Reckelhoff, 2011). Maintenance of cardiovascular health depends, at least in part, on proper body fluid homeostasis. Water and saline intake are two key behaviors that maintain this homeostasis. As is common with many behaviors, there is a sex difference in fluid intake that is largely a function of ovarian hormones. The effects of ovarian hormones are clear from animal studies that report changes in behavior that correspond with cyclic levels of circulating estrogens, specifically estradiol (E2; see Figure 1 for a summary). Laboratory animal studies have revealed influences of ovarian hormones in rodents that have served as important models in this field. These studies will be the focus of the majority of this review.

The modern study of estrogens and fluid intake likely began in 1929 when Richter and Brailey noted that female rats drank less than males did under the same conditions (Richter and Brailey, 1929). Although not mentioned by the authors, the data presented from a single representative female also demonstrate a cyclic change in water intake with lower intakes occurring approximately every four to five days. Much of the subsequent information about the nature of these sex differences would come from rat studies, however, to the best of our knowledge, the earliest report that systematically examined water intake as a function of the ovulatory cycle was performed in 1937 using a single pig-tailed macaque (Krohn and Zuckerman, 1937). This study found that daily water intake decreased during the periovulatory phase, or during the time the authors described as “sexual skin swelling,” in two of three observed menstrual cycles. Later studies in rats confirmed that water intake fluctuates across the estrous cycle with roughly an 8–10% reduction occurring during behavioral estrus (Antunes Rodrigues and Covian, 1963; Tarttelin and Gorski, 1971; Findlay et al., 1979; Danielsen and Buggy, 1980; Eckel et al., 2000) and found that daily water intake increases after ovariectomy (Tarttelin and Gorski, 1971).

Similar changes in saline intake, another component of proper fluid balance, also occur in female rodents. When concentrations of up to 2% saline are offered *ad libitum*, there is also a decrease in intake during behavioral estrus (Antunes Rodrigues and Covian, 1963; Danielsen and Buggy, 1980); however, *ab libitum* intakes of higher, aversive, concentrations of saline have been reported to be unchanged across the cycle (Findlay et al., 1979). Hormone replacement studies in the subsequent decades demonstrated that E2, the most prevalent circulating estrogen in humans and rodents, is sufficient to decrease fluid intake in ovariectomized (OVX) rodents (Spiteri et al., 1980; Jonklaas and Buggy, 1984; Stricker et al., 1991; Scheidler et al., 1994), whereas treatment with progesterone has no effect either on its own or in combination with E2 (Thrasher and Fregly, 1977; Spiteri et al., 1980; Kisley et al., 1999b). These studies highlight the importance of estrogens in mediating fluid intake effects, and highlight a contrast with the control of reproductive behaviors, which involve cooperation of effects of both estrogens and progestins.

Because rodents are prandial drinkers, many studies of estrogen effects on fluid intake are confounded by its anorexigenic effect. One set of studies addressing this used OVX guinea pigs and restriction of either food or water intakes (Czaja et al., 1983). These experiments demonstrated that food and water intakes can be affected by E2 independent of each other. Additional support for an effect of estrogens on fluid intake independent of any concomitant effects on food intake is found in studies using dipsogenic treatment conditions during which food intake is normally absent or minimal. A more comprehensive review of these studies is found elsewhere (e.g., Curtis, 2009; Xue et al., 2013), and the data seem to clearly show that the effects of estrogens on fluid intake persist, even when there is no potential influence of a more direct effect of food intake.

## Sex differences

Sex differences arise from a variety of mechanisms, occurring at different times in development. Although studies of estrogen effects on ingestive behavior largely focus on the behavior in adulthood (i.e., activational effects), it is difficult to understand these effects fully without first understanding how the brain is organized to respond accordingly. Indeed, in adult animals, phasic decreases in water intake are the result of fluctuations in estrogens during the estrous cycle, but the brain must be organized in a way that renders it capable of both generating the cycle and responding to the cyclic hormonal signals. The primary question here is whether or not differences in baseline or stimulated intake are a function of different levels of circulating hormones in the adult or different organization of the responsive nodes during development (or both). For instance, as described above, female rats drink less water than male rats (Richter and Brailey, 1929), but this simple observation does not provide information about whether this is the result of activational or organizational effects. Studies showing decreased intake after E2 provide evidence for an activational effect of estrogens in female rats, but the lack of an effect of E2 in adult male rats (Jonklaas and Buggy, 1984, 1985) suggests that organizational effects also are necessary. Consistent with many other studies of the organizational effect of gonadal hormones, neonatal castration of male rats enables an anti-dipsogenic response to central E2 in adulthood. In turn, neonatal androgen treatment in female pups prevents the decrease in fluid intake that would otherwise be caused by E2 exposure in adulthood (Jonklaas and Buggy, 1985). As such, it appears that both organizing and activating effects of steroid hormones play a role in water intake.

Evaluating organizational and activational sex differences is rather straightforward when considering the controls of water intake, but expanding the discussion to sodium intake is a bit more complicated. Similar to its effect on water intake, phasic E2 decreases saline intake under most conditions. These observations differ from studies of water intake because the response does not appear to be sexually dimorphic; unlike effects of E2 on water intake that only occur in females, it appears that male and female rats both decrease saline intake when treated with E2 (Stricker et al., 1991). This argues that there is no organizational sex difference affecting this function of E2. Paradoxically, however, in spite of having much lower circulating estrogens, males drink less saline under most conditions than do females (Krecek et al., 1972; Wolf, 1982; Sakai et al., 1989; Chow et al., 1992; Flynn et al., 1993; Curtis et al., 2004). For instance, the combination of mild sodium deficiency and the diuretic furosemide reliably stimulates saline intake in both male and female rats. This intake is a function of the increased levels of angiotensin II (AngII) and the sodium loss caused by the treatment. Although the effect is reliable in both sexes, the saline intake observed is twice as much when female rats are used as subjects than it is when male rats are used (Wolf, 1982). This is true using a variety of saline concentrations ranging from 0.5–6% and doses of furosemide (0–10 mg). This initial study was limited, however, because it used intact females and did not control for stage of the estrous cycle (Wolf, 1982), but other studies demonstrate that even when females are OVX, thereby removing the confounding issue of the cycle, intake by the female rats is still greater than it is by male subjects (Chow et al., 1992). Thus, whereas estrogens have a clear anti-dipsogenic and anti-natriorexigenic effect acutely, the higher circulating estrogens in female rats do not generate an overall lower level of saline intake than would be predicted. As such, it appears that there is an underlying sex difference in saline intake that occurs separately from activational effects of ovarian hormones.

The sex difference in baseline saline intake appears to be the result of organizational effects of gonadal hormones. Indeed, the sex difference in saline intake emerges early and can be observed in pups as young as 14 days using renin treatment to stimulate saline intake (Leshem and Epstein, 1989). Gonadectomy during adulthood or 10 days after birth does not mitigate sex difference in saline intake (Krecek et al., 1972; Chow et al., 1992), but castration earlier than 10 days, during an apparent critical window, leads to higher, female-like saline intake in adulthood. In contrast, neonatal testosterone treatment causes females to exhibit lower, male-like levels of *ad libitum* saline in adulthood. Like many organizational sex differences (Arnold and Breedlove, 1985; de Vries and Södersten, 2009), this appears to require aromatization of androgens into estrogens because DHT, a non-aromatizable androgen, does not alter adult intake after neonatal exposure (Chow et al., 1992). Although little is known about the specific organizational effects that lead to these differences, altered taste sensitivity appears to be one endpoint of these organizational effects because taste reactivity testing and electrophysiological recordings suggest that different gustatory sensitivity contribute to the observed sex differences (Flynn et al., 1993; Curtis and Contreras, 2006).

## Role of estrogens in the double-depletion hypothesis of thirst and salt appetite

The double-depletion hypothesis of water and salt intake, first described by Fitzsimons (1973) and Epstein (1973) argues that fluid intake in mammals is stimulated by alterations of either the intracellular or the extracellular fluid compartments, that altering the intracellular space is detected by a separable mechanism from that which detects changes in the extracellular space, and that the behavioral response to the dehydration of the intracellular compartment differs from the response to dehydration of the extracellular compartment. Intracellular dehydration, generally caused by increased solute in the extracellular space that dehydrates the intracellular compartment by osmosis, is detected by osmoreceptors that are critical for the onset of fluid intake, but the behavioral response involves consumption of water with an apparent inhibition of saline intake. Loss of fluid from the extracellular compartment results in hypovolemia without affecting the concentration of the remaining fluid. Accordingly, perturbations of this type do not engage osmoreceptive elements of the CNS. Instead, detection of hypovolemia occurs largely by volume receptors in the periphery; specifically by vascular and renal baroreceptors that communicate with the CNS through vagal afferents and by increased activity of the renin-angiotensin system. Unlike the behavioral response to intracellular fluid loss that selectively increases water intake, loss of fluid from the extracellular space increases intake of both water and sodium. The different stimuli, detection mechanisms, and behavioral responses allow for the use of different models of fluid intake in order to determine if things that affect the controlling elements, for example estrogens, are selective for one or the other, or are more general.

Estrogens appear to have minimal influence on drinking in response to osmotic stimuli. Water intake induced by subcutaneous hypertonic saline, for example, does not change across the estrous cycle and is not influenced by acute or chronic s.c., intramuscular (i.m.) or intracerebroventricular (i.c.v.) E2 treatment in OVX rats (Findlay et al., 1979; Jonklaas and Buggy, 1984; Krause et al., 2003; Mecawi et al., 2008). There are, however, two reports from the 1970s demonstrating that chronic s.c. treatment of E2 decreased water intake induced by hypertonic saline in intact cycling rats (Thrasher and Fregly, 1977, 1978). Others using more physiologically relevant approaches have attempted to replicate this effect, but have had little success (Jonklaas and Buggy, 1984; Krause et al., 2003; Mecawi et al., 2008), leading to the sense that estrogens do not actually impact the amount of water consumed after an osmotic challenge. Although estrogens do not seem to influence the volume consumed under these conditions, careful behavioral analyses have, indeed, shown subtle estrogen-mediated differences in water intake. For example, the latency to drink after an intravenous infusion of hypertonic saline is decreased in OVX female rats treated s.c. with acute E2 (Jones and Curtis, 2009). A follow up study, however, showed that hyperosmolality-induced neuronal activation in the area postrema, paraventricular nucleus (PVN), and rostral ventrolateral medulla was decreased in OVX female rats treated with acute s.c. E2 (Jones et al., 2012). The authors of this study suggested that this apparent paradox (the more rapid water intake in the face of a reduced neural response) may reflect either a disinhibition of water intake or a function of changes in a central pressor response. Indeed, increases in blood pressure inhibit fluid intake (Klingbeil et al., 1991; Thunhorst et al., 1993) and estrogens have hypotensive effects in a number of systems (Bachmann et al., 1991). Accordingly, it seems equally plausible that the more rapid water intake is caused by E2-induced changes in cardiovascular tone. Nevertheless, it seems clear that estrogens can play at least an indirect role in water intake after osmotic stimuli.

Estrogens have a more pronounced and clearer role in hypovolemic fluid intake. Because engagement of the renin-angiotensin system is a common feature of models of hypovolemia, injections of AngII are often used to study hypovolemic fluid intake. Intakes of both water and saline stimulated by i.c.v. AngII vary across the estrous cycle with lowest intakes on the day of estrus (Findlay et al., 1979; Danielsen and Buggy, 1980). This is, however, dependent on the concentration of saline provided and seems to be more observable using more palatable concentrations of saline; for example, intake of 1.8% saline was affected by cycle stage, but intake of 2.7% saline was not (Findlay et al., 1979; Danielsen and Buggy, 1980). A cyclic change alone does not demonstrate a role for estrogens in this observation, but several additional findings strongly support it being dependent on ovarian estrogens. First, the cyclic changes in i.c.v. AngII-stimulated intakes are lost after OVX (Findlay et al., 1979). Second, E2 treatment (chronic or acute s.c., or acute i.c.v.) dose-dependently decreases fluid intake after AngII (i.c.v. or intraperitoneal ;i.p.) in OVX rats (Fregly, 1978; Fregly and Thrasher, 1978; Jonklaas and Buggy, 1984; Kisley et al., 1999b). Third, the effect of E2 is blocked by administration of an antiestrogen (Kisley et al., 1999b). Unlike some estrogen-dependent behaviors, progesterone does not appear to modify these effects of E2 (Thrasher and Fregly, 1977; Spiteri et al., 1980; Kisley et al., 1999b). Accordingly, the data strongly support a role for endogenous estrogens, most likely attributable to E2, in the control of saline intake.

Although AngII has been used to model hypovolemia, the response to true hypovolemia involves more than a simple rise in AngII activity. Accordingly, before concluding that estrogens affect hypovolemia-induced fluid intake, we must consider other models of hypovolemia. Indeed, data using other ways to model hypovolemia corroborate the role of estrogens in hypovolemic fluid intake. For instance, fluid intake after treatment with the β-adrenergic agonist isoproterenol is decreased by estrogens. Cyclical variations in isoproterenol sensitivity are found across the estrous cycle, with the lowest isoproterenol-induced intake observed during estrus (Kucharczyk, 1984). Either E2 treatment (acute or chronic s.c.) decreases water and saline intake stimulated by isoproterenol in OVX rats (Thrasher and Fregly, 1977, 1978; Fregly, 1978; Krause et al., 2003; Jones and Curtis, 2009). Thus, drinking after β-adrenergic agonist treatment appears to be subject to the same influence of estrogens as is drinking after AngII and these findings can be further generalized to other models of hypovolemia. Water and saline intakes stimulated by treatment with the diuretic furosemide, either alone or in combination with captopril, are less robust on the day of estrus than they are when the treatments are given on diestrus. Moreover, intake stimulated using this treatment is decreased by s.c. E2 in OVX rats (Mecawi et al., 2008; Dalmasso et al., 2011). Chronic s.c. E2 treatment also decreases water intake stimulated by renin-treatment in OVX rats (Thrasher and Fregly, 1977). Finally, water restriction or maintenance on a sodium deficient diet stimulates less intake when OVX rats are treated with acute or chronic s.c. E2 than it does without E2 (Stricker et al., 1991; Krause et al., 2003; Mecawi et al., 2008). In contrast to the more nuanced and subtle effects of estrogens on water intake after osmotic stimuli, the effects of estrogens on hypovolemic fluid intake are more clear.

## Estrogen receptors

Estrogen receptor (ER) is classically described as a nuclear receptor that acts as a ligand-dependent transcription factor. The initial view that this occurs through a single receptor type has been expanded to include two nuclear receptors, ERα and ERβ. Indeed, estrogens binding at ERα and/or ERβ cause the formation of hetero- or homodimers of these receptor proteins and initiates recruitment of coactivators /corepressors that increase or decrease transcription (O’Lone et al., 2004; Heldring et al., 2007). The classical view has been further expanded by the identification of novel estrogen binding sites more closely associated with or embedded in the cell membrane. Novel proteins responsible for a subset of these membrane-associated binding sites have been identified and named GPR30, ERX, and Gq-mER and there is now documentation that both ERα and ERβ can localize to the plasma membrane and act as surface receptors (Micevych and Dominguez, 2009; Micevych and Kelly, 2012). When activated by estrogens, these receptors stimulate intracellular signaling cascades that can change the excitability of neurons through the rapid activation of kinases and increases in calcium. In addition, these receptors influence gene expression though intracellular signaling cascades that activate transcription factors such as cAMP response element binding protein (CREB; Vasudevan and Pfaff, 2007; Micevych and Dominguez, 2009). As such, there are several mechanisms through which estrogens can act and means to affect gene expression.

There are ERs in all brain regions implicated in the control of fluid intake although not all nuclei express each ER subtype (Shughrue et al., 1997; Shughrue and Merchenthaler, 2001; Brailoiu et al., 2007). For example, ERα appears to be the only subtype expressed in the subfornical organ (SFO) whereas ERβ and GPR30, but not ERα, are expressed in the PVN and supraoptic nucleus (SON; Shughrue et al., 1997; Shughrue and Merchenthaler, 2001; Brailoiu et al., 2007). To date there have been very few studies examining the roles of ER subtypes in fluid intake in female subjects.

### ER subtypes and fluid intake

Attempts to determine the role of individual ER subtypes in fluid intake have been complicated by the variety of receptors (ERα, ERβ, GPR30, etc.) and their cellular distribution (nuclear vs. membrane-associated). Our laboratory has started to address this question empirically. As a first step, we tested for intake effects of membrane-associated ER subtypes. These studies rely on information from others showing that estrogen effects on fluid intake often take hours or days to become observable after the treatment (Kisley et al., 1999b; Krause et al., 2003). In a study specifically designed to test for the rapid behavioral effects of acute s.c. E2 on fluid intake, there was no change in short term (15 min) water intake stimulated by isoproterenol in OVX rats. The earliest observable effects of E2 were not apparent until 24 h after treatment (Graves et al., 2011). The delayed response is consistent with a genomic effect of estrogens, but, as described above, either direct action of nuclear ER or indirect action of membrane ER can influence gene transcription. As such, we performed experiments to determine if the observed effects involved only nuclear receptors or if membrane ER subtypes play a role. Specifically, we treated OVX rats i.c.v. with an E2-BSA conjugate, which cannot enter the cell and is therefore limited to activating membrane receptors, and found decreases in overnight water intake (Santollo et al., 2013). Furthermore, analysis of licking patterns suggests that E2 effects on intake are due to changes in both post-ingestive and orosensory aspects of water intake, but when only membrane ER was activated, the decrease in water intake was mediated only by a change in post-ingestive signals. This suggests that membrane ER is involved in modulating satiety signals associated with water intake and the difference in the effects of E2 and the E2-BSA argues that the different populations of ER (membrane-associated and nuclear) play separable roles in controlling fluid intake. Although these studies support a role for membrane-associated ER subtypes in the control of overnight water intake, they do not identify a specific subtype involved. In addition, it is possible that the ER populations mediating E2’s anti-dipsogenic effect on overnight water intake are different from the ER populations mediating its effect on stimulated water intake. Indeed, ongoing and future studies will be important to address these questions and gain a better understanding of the role of ER subtypes in the control of fluid intake.

### Fluid balance and ER expression

Although the estrogens appear to primarily affect fluid intake after hypovolemic challenges, perturbations of either intracellular or extracellular fluid compartments appear to affect ER expression. Major changes in ERα and ERβ expression are highlighted here but for an in depth review please see Somponpun (2007). For instance, ERβ mRNA in the SON is increased by salt-loading and decreased by hypo-osmolality caused by s.c. infusions of the vasopressin analog, 1-desamino-[8-D-arginine] vasopressin (Somponpun and Sladek, 2003). Similarly, ERβ is decreased in both the SON and PVN after 48 h water deprivation (Somponpun et al., 2004b) and after polyethylene glycol-induced hypovolemia (Somponpun and Sladek, 2004). Interestingly, ablations of the anteroventral third ventricle region (AV3V) eliminate the effect of water deprivation in the SON, but not the PVN (Somponpun et al., 2004b). Although the effects in these studies were selective to ERβ, other work has shown water deprivation-induced increases in SFO ERα (Somponpun et al., 2004a). Unfortunately, to the best or our knowledge, all published studies examining these effects to date have used male rats, so the relevance to the anti-dipsogenic and anti-natriorexigenic responses to estrogens in females is unclear. Future studies will be important to test for any sex differences in the response and to test for causal relationships between the changes in receptor expression and behavior.

## Hypothalamic site of action

Studies examining behavioral and electrophysiological responses to AngII have identified multiple hypothalamic nuclei and nearby forebrain structures that are important for anti-dipsogenic and anti-natriorexigenic effect of estrogens. Included in this list are the SFO, lateral hypothalamus (LH), PVN, SON, median preoptic area (MnPO), medial preoptic area (mPOA), and organum vasculosum of the lamina terminalis (OVLT). Importantly, at least one ER subtype is expressed in each of these brain areas and many express more than one type of ER (Shughrue et al., 1997; Shughrue and Merchenthaler, 2001; Brailoiu et al., 2007). Additionally, sites such as the SON, that do not express certain ER subtypes, specifically ERα, can respond to the relevant signals through projections from ERα-expressing neurons in surrounding nuclei (Voisin et al., 1997).

There is ample data demonstrating that the SFO plays a major role in the dispogenic effect of E2 via projections to, and input from, other hypothalamic nuclei. AngII injection directly into the SFO stimulates water intake that can be attenuated by chronic s.c. E2 treatment in OVX rats (Fujisawa et al., 2001; Tanaka et al., 2002, 2003). Co-localization of ERα and angiotensin type 1 receptor (AT1R) in SFO neurons provides further evidence for the SFO as an important site of action (Rosas-Arellano et al., 1999). This hypothesis is additionally supported by studies of neural activity. For instance, in OVX rats the effect of intra-carotid AngII on neurophysiological recordings of SFO neurons is attenuated by chronic s.c. E2 treatment (Ciriello and Roder, 2013) and isoproterenol-induced Fos-immunoreactivity in the SFO is decreased by acute s.c. E2 treatment (Krause et al., 2006).

Although the SFO is a strong candidate structure for an interaction between AngII and estrogens, it clearly does not act in isolation and is, instead, part of a broader distributed circuit in the hypothalamus. Relevant connections between the SFO and the LH, PVN, and MnPO appear important for the mediation of anti-dipsogenic effects of estrogens. For instance, in OVX rats LH injections of AngII stimulate water intake and this is attenuated by chronic s.c. E2 (Fujisawa et al., 2001). This effect likely involves the efferent projections to the SFO (Lind et al., 1984) because water intake stimulated by AngII treatment in the LH is attenuated by pretreatment with an angiotensin receptor antagonist, saralasin, in the SFO. Water intake was further attenuated in OVX rats treated with chronic s.c. E2, suggesting that AngII-responsive neurons that project from the LH to the SFO are inhibited by E2 (Fujisawa et al., 2001). Furthermore another study in OVX rats demonstrated that SFO neurons showed less excitation after iontophoretically applied AngII and electrical stimulation of the LH after chronic s.c. E2 treatment compared to controls (Tanaka et al., 2001).

Behavioral and electrophysiological studies demonstrate that the PVN acts downstream of the SFO in mediating an anti-dipsogenic effect of estrogens. Water intake stimulated by AngII treatment in the SFO was attenuated by pretreatment with the AngII antagonist saralasin into the PVN. Water intake was further attenuated in OVX rats that were treated chronically with s.c. E2 (Tanaka et al., 2002). Furthermore, in OVX rats acute s.c. E2 has been reported to decrease spontaneous firing rate and increase the absolute refractory period in SFO neurons which have been antidromically activated by electrical stimulation of the PVN (Tanaka et al., 2001). In this same study, acute s.c. E2 treatment in OVX rats decreased AngII stimulated neuronal excitation of PVN activated SFO neurons (Tanaka et al., 2001). Similar studies demonstrated that pretreatment with saralasin in the MnPO decreased water intake stimulated by AngII-treatment in the SFO, which is further attenuated by chronic s.c. E2 treatment in OVX rats (Tanaka et al., 2003). Finally, in OVX rats there is a decrease in neuronal firing after AngII in SFO neurons antidromically activated by SON stimulation after treatment with chronic s.c. E2 (Ciriello and Roder, 2013). Taken together these data show that the SFO, LH, PVN, MnPO and SON are all part of the estrogen-sensitive circuitry that are involved in fluid intake.

**Figure 2:**
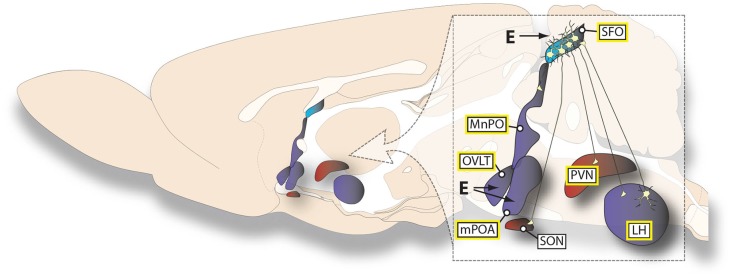
**Summary of estrogen effects on AngII-sensitive neural circuits**. The illustration summarizes the findings reviewed here regarding the specific hypothalamic connections that are affected by both estrogens and AngII. The different colors represent the primary ER subtype expressed in a given structure (ERα, blue; both ERβ and GPR30, red; both ERα and ERβ, purple). Structures in which AngII can act directly to stimulate water intake are highlighted by a yellow border around the label. The lack of a connection or a site of action depicted should not be viewed as evidence for the absence of any projection or effect, but simply reflects the lack of experimental support for its existence.

In addition to the brain regions highlighted above that have been studied with respect to their connections with the SFO, the mPOA and OVLT also appear to be involved in mediating anti-dipsogenic and anti-natriorexigenic effects of estrogens, perhaps independent of any connections these structures have with the SFO. AngII injections directly into the mPOA stimulate water intake and this is attenuated on the day of estrus (Kucharczyk, 1984). This may be, at least in part, due to direct effects of estrogens on mPOA neurons because acute E2 injections directly into the mPOA of OVX rats decrease water intake stimulated by lateral ventricle injections of AngII (Jonklaas and Buggy, 1985). Indeed, this finding could be seen as evidence that some of the effects of less focused treatments with E2 act at the mPOA to reduce intake. Similarly, E2 injection directly into the region that comprises the front wall of the third ventricle decreases both AngII-stimulated and overnight water intake in OVX rats (Jonklaas and Buggy, 1984); however, this effect is likely more specific to cells in the OVLT than in the rest of the ventral *lamina terminalis* because intake after AngII injections directly into the MnPO, which lies dorsal to the OVLT, is not influenced by chronic s.c. E2 treatment in OVX rats (Tanaka et al., 2003). Natriorexigenic treatments such as furosemide increase the number of Fos-positive cells in the OVLT and the magnitude of this effect is attenuated in rats during estrus. Surprisingly, the magnitude of the effect is also attenuated in OVX rats, even though the predicted effect of OVX would be the opposite of what is observed. Furthermore, acute s.c. E2 treatment in OVX rats completely eliminated the furosemide-induced increase in Fos in the OVLT (Dalmasso et al., 2011). Importantly, these responses seem to be anatomically selective because chronic E2 infusion into the ventromedial hypothalamus (VMH) of OVX rats does not change i.c.v. AngII-induced water intake (Jonklaas and Buggy, 1985), even though the VMH contains dense ER expression (Shughrue et al., 1997). Together these studies demonstrate an involvement of multiple hypothalamic nuclei in mediating anti-dipsogenic/anti-natriorexigenic effects of estrogens (summarized in Figure 2).

## Estrogen-peptide interactions

Localization of the action of estrogens provides clues into the possible mechanism(s) underlying the anti-dipsogenic/anti-natriorexigenic effect. Because the hypothalamus and nearby structures are sites where estrogens influences fluid intake, the neuropeptides that act or are expressed in these areas are possible targets of estrogens. Indeed, a number of peptides and their receptors in these areas are influenced by estrogens and this likely contributes to these changes in fluid intake. Without attempting to generate an inclusive list, three peptide systems that are important for fluid homeostasis are highlighted here.

### Angiotensin

Many studies described already in this review have shown a clear effect of estrogens on AngII-induced fluid intake. Based on this interaction, additional studies have investigated changes in the angiotensin receptor, specifically the AT1R, as a potential mechanism for the observed behavioral changes. Indeed, in OVX rats acute s.c. E2 decreased AngII binding in large blocks of tissue that included the thalamus, septum and preoptic area, without any observable change in tissue blocks that included the hypothalamus, midbrain or olfactory area (Jonklaas and Buggy, 1985). The anatomical locus of this change was refined by later studies to include acute s.c. E2-induced changes in AT1R in the SFO, but no changes in the OVLT, MnPO, suprachiasmatic nucleus, SON, PVN or median eminence were found (Kisley et al., 1999a). Studies that removed endogenous estrogens by OVX supported the previous results by showing OVX-induced increases in AT1R binding in the SFO, and also found increases in the SFO, PVN and MnPO (Dean et al., 2005). Whether the change in binding is a function of altered receptor affinity or downregulation of the receptor remains unclear, but decreased AT1R mRNA in relatively large blocks of tissue was also reported in one study (Kisley et al., 1999a), suggesting a primary effect on receptor availability. The questions asked by these studies have been revisited recently using more refined approaches that examined AT1R expression and binding in discrete nuclei in OVX rats and found a decrease in AT1R mRNA after acute s.c. E2 treatment in the SFO and a decreases in AT1R binding in the SFO, OVLT, PVN and MnPO after chronic s.c. E2 treatment (Dean et al., 2005; Krause et al., 2006). It is important to note that in the study by Krause et al. weight loss caused a reduction in AT1R expression in the SFO and a reduction in the dipsogenic response, both of which were reduced by a similar magnitude as the decrease caused by E2 (Krause et al., 2006). Accordingly, it remains unclear if the observed change in AT1R expression is secondary to the weight loss caused by E2 treatment. Regardless of the more proximal cause, these studies provide strong support that estrogens decrease both binding and expression of AT1R. The complete story is further complicated, however, by reports of *increased* angiotensin binding and/or mRNA in the locus coeruleus, MnPO, SFO and ARC when OVX rats were given acute s.c. E2 and progesterone (Donadio et al., 2005, 2006). Based on these data, one might conclude that E2 alone is quite different in its effects from the combination of E2 and progesterone, but the behavioral relevance of this finding would be questionable because studies of progesterone effects in fluid intake have not revealed any obvious contribution (Thrasher and Fregly, 1977; Spiteri et al., 1980; Kisley et al., 1999b).

In addition to effects on AT1R, estrogens also influences other parts of the renin-angiotensin system. Many of these effects occur in the periphery and are, therefore, outside the of the present focus on the CNS (for review of these changes, please see Kuroski de Bold, 1999; Fischer et al., 2002), but the more recent recognition of a central renin-angiotensin system (for review, see McKinley et al., 2003), offers an important target for future investigation. Indeed two recent studies demonstrate that angiotensin converting enzyme (ACE) expression and binding in the lamina terminalis increases after OVX with acute i.c.v. or chronic s.c. E2 replacement (respectively) restoring levels to those observed in intact females (Dean et al., 2005; Xue et al., 2014). Future studies are required to further investigate these changes and to isolate components of the central renin-angiotensin system as a target of estrogens.

### Oxytocin

Oxytocin inhibits saline intake and appears to act as a brake to prevent saline intake when water intake is stimulated by intracellular dehydration (Blackburn et al., 1992, 1993). This is an important function because saline intake during intracellular dehydration would further exacerbate the deficit, and why it is physiologically important that the effect of oxytocin is limited to saline intake and does not generalize to water intake (Blackburn et al., 1992, 1993). The mechanism of this inhibition is unclear, but some clues may arise from early studies of reproductive behaviors and the interactions between oxytocin and estrogens in hypothalamic regions such as the VMH (Ivell and Walther, 1999). In cycling female rats, oxytocin receptor (OTR) expression in the VMH fluctuates with the estrous cycle and is at the highest level during proestrus (Bale et al., 1995a). Acute s.c. E2 increases OTR expression in the VMH and increases OTR binding in OVX rats (Bale and Dorsa, 1995; Bale et al., 1995b). Estrogens also affect the oxytocin system in structures known to be involved in fluid homeostasis. Multiple studies have demonstrated that oxytocin mRNA and immunoreactivity in the SON and PVN are increased by chronic s.c. E2 treatment of OVX rats (Jirikowski et al., 1988; Caldwell et al., 1989; Crowley et al., 1995). A later report in OVX rats, however, showed that oxytocin mRNA in the PVN is decreased after E2 treatment, whereas expression was unchanged in the SON (Shughrue et al., 2002). The latter study used a single acute injection of E2, making the different results potentially reflective of differences in the duration of E2 action (Shughrue et al., 2002). Nevertheless, these changes are likely mediated by either ERβ or GPR30 because double-labeling approaches reveal co-expression of these proteins in cells in both the PVN and SON (Hrabovszky et al., 1998; Shughrue et al., 2002; Brailoiu et al., 2007; Sakamoto et al., 2007).

In addition to more direct estrogen-induced changes in oxytocin or its receptors, the ability of other stimuli that normally affect oxytocin, such as an osmotic challenge, is affected by estrogens. Specifically, treatment with hypertonic saline increases plasma oxytocin during the late afternoon of estrus and diestrus, and E2 enhances the increase in plasma oxytocin after hypertonic saline exposure in OVX rats. On the other hand, oxytocin in the PVN is increased by hypertonic saline administration during proestrus, but not during diestrus or estrus (Caligioni and Franci, 2002), arguing that the measures of plasma and PVN oxytocin are not necessarily consistent. Interpretation of some of these results is clouded by overlapping and inconsistent nomenclature, however, presenting the possibility that many of the things said to occur during proestrus are actually occurring during what we refer to as behavioral estrus (for review of this nomenclature issue, please see Becker et al., 2005). Nevertheless, the concept that oxytocinergic tone is increased by estrogens, and the evidence for inhibition of saline intake by oxytocin, are collectively consistent with the hypothesis that estrogens decrease saline intake, at least in part, by increasing activity in oxytocin responsive neurons. Other studies, however, complicate this otherwise parsimonious explanation. As noted above, estrogens have only subtle effects on ingestive responses to intracellular (osmotic) stimuli, but oxytocin is part of the response to osmotic perturbation and oxytocin is affected by estrogens. If any oxytocin that participates in the control of fluid intake is similarly affected, then OVX should have a noticeable effect on the behavioral response to hypertonic saline. As such, the relevance of oxytocin and its interactions with estrogens remain poorly understood factors in the control of fluid intake.

### Vasopressin

Although vasopressin does not influence fluid intake directly, its secretion is stimulated in parallel with fluid intake and it is clearly critical in the maintenance of body fluid homeostasis (Daniels and Fluharty, 2009). Given the effects of estrogens on fluid intake, and that ER subtypes are expressed within vasopressin expressing neurons (Hrabovszky et al., 1998, 2004; Brailoiu et al., 2007), it may not be surprising to find concurrent effects of estrogens on the vasopressin system. The literature, however, is full of conflicting reports of sex differences in the vasopressin system and the relevant effects of estrogens (for review, please see Sladek and Somponpun, 2008). Whereas some have not found E2-related changes in blood vasopressin by E2 (Crofton et al., 1985; Wang et al., 1996), others have reported that baseline levels of vasopressin increase during early proestrus in intact rats (Skowsky et al., 1979). The differences in these reports could be due to the specific timing of the obtained measurements and the light cycles used by the investigators. Other studies have found that removal of endogenous estrogens by OVX decreases blood vasopressin (Peysner and Forsling, 1990) and that circulating vasopressin is increased by low doses of s.c. E2 but decreased by high doses of s.c. E2 (Skowsky et al., 1979; Peysner and Forsling, 1990). The increase in vasopressin mediated by the lower doses of E2 is consistent with electrophysiological recordings finding that chronic s.c. E2 doubles the discharge rate of vasopressin secreting neurons in the SON of OVX rats (Akaishi and Sakuma, 1990), but studies using hypothalamo-neurohypophyseal explants found that vasopressin release stimulated by glutamate agonist or hypertonic conditions is inhibited after bath application of E2 (Swenson et al., 1998). In light of the seemingly conflicting results, it seems especially important to consider the effects in the whole animal. Indeed, studies of this nature find that OVX reduces the pituitary vasopressin release and expression that is normally stimulated by either osmotic or hypovolemic challenges and that this reduction is prevented by acute or chronic s.c. E2 replacement (Crowley and Amico, 1993; Hartley et al., 2004). This is consistent with the finding that acute s.c. E2 increases i.c.v. AngII-induced Fos expression in PVN vasopressin neurons of OVX rats (Kisley et al., 2000). Accordingly, the data from these studies suggests opposite effects of estrogens on fluid intake and the vasopressin system. It is tempting to speculate that the opposite direction of the effects (E2 decreasing intake while often increasing vasopressin) reveals an interesting way that the system can maintain homeostasis in the face of decreased fluid intake. While intake is reduced by estrogens, diuresis is simultaneously reduced by the increases in vasopressin, allowing the animal to drink less and still maintain adequate hydration. This is coupled with an increase in the anti-diuretic potency of vasopressin in females during estrus (Wang et al., 1995). Thus, these systems appear to respond to estrogens in opposite directions to maintain homeostasis.

## Concluding remarks

Estrogens have obvious effects on behavior and health and regulation of fluid intake is particularly critical for cardiovascular health. In addition to this more direct translational relevance, studies of estrogens on fluid intake serve as an important basic model for understanding broader questions related to behavioral effects of steroid hormones and steroid-peptide interactions. This review has focused on previous studies examining the effects of estrogens on water and salt intakes, and clearly demonstrates the sparsity of studies in this area while highlighting the many open questions that remain. It is especially notable that many studies in this subfield use only male subjects and often make erroneous assumptions that the conclusions drawn from these studies can be extended to females. Indeed, direct comparisons have repeatedly revealed striking contrasts. This issue is additionally complicated by the discrepancies that can arise when attempting to translate basic research to clinical application. This is not to say that a translational approach is impossible. Indeed, there are studies demonstrating cycle-related changes in fluid balance in humans. For example, the amount of hypertonic saline needed to stimulate water intake decreases in women during the luteal phase, (Vokes et al., 1988) (for recent reviews of the human literature, see Stachenfeld, 2008; Wenner and Stachenfeld, 2012), but further studies are needed, both at the basic and translational levels. We are cautiously optimistic, however, that the recent strategic plan of the National Institutes of Health (Pinn et al., 2010) will help to address these important issues.

## Conflict of interest statement

The authors declare that the research was conducted in the absence of any commercial or financial relationships that could be construed as a potential conflict of interest.
